# EPRS is a critical regulator of cell proliferation and estrogen signaling in ER^+^ breast cancer

**DOI:** 10.18632/oncotarget.11870

**Published:** 2016-09-06

**Authors:** Igor Katsyv, Minghui Wang, Won Min Song, Xianxiao Zhou, Yongzhong Zhao, Sun Park, Jun Zhu, Bin Zhang, Hanna Y. Irie

**Affiliations:** ^1^ Medical Scientist Training Program, Icahn School of Medicine at Mount Sinai, New York, NY 10029, USA; ^2^ Icahn Institute for Genomics and Multiscale Biology, Icahn School of Medicine at Mount Sinai, New York, NY 10029, USA; ^3^ Department of Genetics and Genomic Sciences, Icahn School of Medicine at Mount Sinai, New York, NY 10029, USA; ^4^ Division of Hematology and Medical Oncology, Department of Medicine, Icahn School of Medicine at Mount Sinai, New York, NY 10029, USA; ^5^ Department of Oncological Sciences, Tisch Cancer Institute, Icahn School of Medicine at Mount Sinai, New York, NY 10029, USA

**Keywords:** breast cancer, ER+, EPRS, gene networks

## Abstract

Aminoacyl tRNA synthetases (ARSs) are a class of enzymes with well-conserved housekeeping functions in cellular translation. Recent evidence suggests that ARS genes may participate in a wide array of cellular processes, and may contribute to the pathology of autoimmune disease, cancer, and other diseases. Several studies have suggested a role for the glutamyl prolyl tRNA synthetase (*EPRS*) in breast cancers, although none has identified any underlying mechanism about how *EPRS* contributes to carcinogenesis. In this study, we identified *EPRS* as upregulated in estrogen receptor positive (ER+) human breast tumors in the TCGA and METABRIC cohorts, with copy number gains in nearly 50% of samples in both datasets. *EPRS* expression is associated with reduced overall survival in patients with ER+ tumors in TCGA and METABRIC datasets. *EPRS* expression was also associated with reduced distant relapse-free survival in patients treated with adjuvant tamoxifen monotherapy for five years, and *EPRS*-correlated genes were highly enriched for genes predictive of a poor response to tamoxifen. We demonstrated the necessity of *EPRS* for proliferation of tamoxifen-resistant ER+ breast cancer, but not ER- breast cancer cells. Transcriptomic profiling showed that *EPRS* regulated cell cycle and estrogen response genes. Finally, we constructed a causal gene network based on over 2500 ER+ breast tumor samples to build up an *EPRS*-estrogen signaling pathway. *EPRS* and its regulated estrogenic gene network may offer a promising alternative approach to target ER+ breast cancers that are refractory to current anti-estrogens.

## INTRODUCTION

ARSs are enzymes that charge tRNAs with their cognate amino acids. In higher eukaryotes, however, ARSs contain additional domains that allow them functions beyond their canonical roles in translation, and several studies have reported dysregulation of ARSs and their noncanonical functions in disease [1]. It is unclear, however, if this dysregulation contributes to or is a byproduct of disease-driving processes.

The glutamyl prolyl tRNA synthetase (*EPRS*) was identified as a tumor immunogen in human breast and gastrointestinal cancers [2], as well as in spontaneous tumors in neu-transgenic mice [3]. Kim et al. identified EPRS copy number gains in lung, esophageal, hepatocellular, skin and breast cancers tumors [4]. *EPRS* has also been implicated in engrailed 1 (EN1)-mediated survival of triple-negative breast cancer cells [5], but not in ER+ breast cancers. EPRS protein is comprised of N-terminus glutamyl-tRNA synthetase (ERS) domain, a C-terminus prolyl-tRNA synthetase (PRS) domain, and linker composed of three 50-amino-acid-long WHEP domains. While the ERS and PRS domains carry out the canonical and functions of EPRS – aminoacylation of cognate tRNAs – the WHEP domains have been implicated in transcript-specific regulation of translation, particularly of the genes ceruloplasmin and VEGFA [6, 7]. While this suggests *EPRS* may play a role in tumor angiogenesis, a functional link between EPRS and angiogenesis has not been established in tumors.

In this study, we examined the role of *EPRS* in the pathogenesis of ER+ breast cancers, which comprise over 70% of breast cancers and which are treated primarily with anti-estrogen drugs, such as tamoxifen. We showed elevated *EPRS* transcript levels in ER+ breast cancer samples compared to adjacent normal breast tissue, and *EPRS* copy number gain in ER+ breast cancers. *EPRS* expression was associated with reduced overall survival in patients with ER+, but not ER- breast cancers and was associated with reduced distant relapse-free survival in patients treated with tamoxifen adjuvant monotherapy. *EPRS*-correlated transcripts in ER+ breast cancer samples were highly enriched for genes predictive of relative resistance to tamoxifen, and *EPRS* inhibition induced a G1/S arrest in tamoxifen-resistant MCF7 cells, but not in ER- breast cancer cells. Using RNA-sequencing and Bayesian network inference, we demonstrated that *EPRS* is a critical regulator of *ERα* expression and activity. Our findings raise the possibility that *EPRS* inhibition may be an alternative approach to suppressing the growth of ER+ breast cancers refractory to tamoxifen treatment. To our knowledge, we are the first to implicate *EPRS* in ER+ breast cancer and the first to describe an underlying, tumor cell-intrinsic mechanism through which *EPRS* may contribute to ER+ breast tumorigenesis.

## RESULTS

### *EPRS* is upregulated in ER+ breast cancers and is associated with reduced overall survival

**Figure 1:**
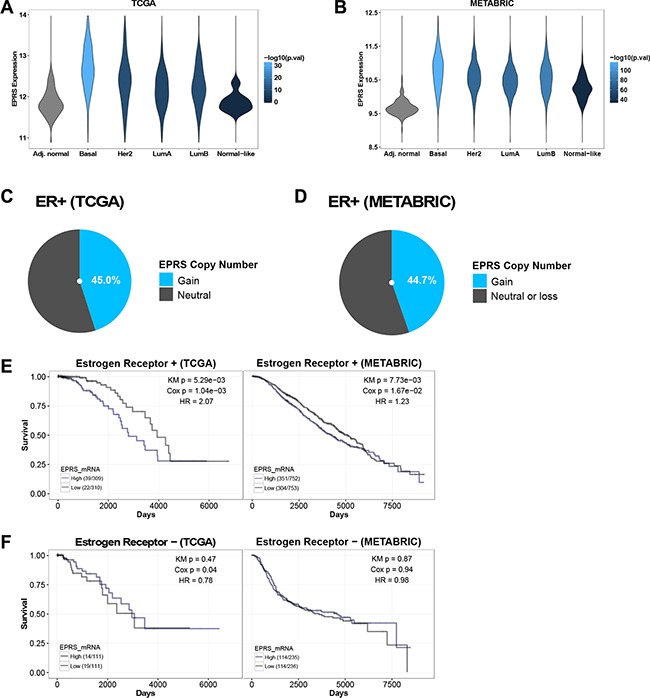
*EPRS* is upregulated in ER+ breast cancers, and is associated with worse outcomes in ER^+^ tumors *EPRS* is upregulated in all breast cancer subtypes compared to adjacent normal breast in TCGA (**A**) and METABRIC (**B**) cohorts. Violin plot color represents significance of student's *t*-test comparing each subtype with adjacent normal breast. Fraction of TCGA ER+ breast cancer samples in COSMIC (**C**) and fraction of METABRIC (**D**) ER+ samples with *EPRS* copy number gains. High *EPRS* expression is associated with worse prognosis in patients with ER+ (**E**) but not ER^−^ (**F**) breast cancer in TCGA and METABRIC cohorts. “High” and “Low” refer to stratification of patients by median *EPRS* expression: upper 50% were called “high,” lower 50% were called “low.” Numbers in parenthesis (e.g. 39/309) are [number of patients who died]/[number of patients at risk].

We compared expression of ARS genes in ER+ tumor and adjacent normal samples in TCGA and METABRIC cohorts (Supplementary Figure S1). Twenty-eight of the 37 genes were differentially expressed in the TCGA cohort and 35 in the METABRIC cohorts at an FDR cutoff of 0.05 (Supplementary Figure S1). Meta-analysis of differential expression in the two cohorts, by summing −log10 *p*-values, identified *EPRS* as the most significantly differentially expressed ARS (TCGA: logFC = 0.39, student's *t*-test *p* = 6.70e-14; METABRIC: logFC = 0.80, student's *t*-test *p* = 1.04e-87) (Supplementary Figure S1) (Figure 1A and 1B). Interestingly, among ER+ tumors, *EPRS* mRNA expression was higher in the relatively more endocrine therapy resistant Luminal B tumors compared to Luminal A tumors in TCGA (logFC = 0.10, student's *t*-test *p* = 0.05) and METABRIC (logFC = 0.10, student's *t*-test *p* = 4.78e-05) cohorts (Figure 1A and 1B). *EPRS* expression is additionally upregulated in basal, Her2, and normal-like breast tumors (Figure 1A and 1B).

*EPRS* maps to chromosome 1q41, amplifications of which frequently occur in breast cancers [8, 9] and are associated with bone metastasis [10]. Single nucleotide polymorphisms in 1q41 have been linked in genome-wide association studies with increased risk of colorectal cancer [11]. Using COSMIC, we identified EPRS copy number gains in 68 of 151 (45.0%) ER+ breast cancer samples from the TCGA cohort (Figure 1C); *EPRS* copy number gains are present in 672 of 1505 (44.7%) of METABRIC ER+ breast cancer samples (Figure 1D). *EPRS* copy number gains may thus partially account for elevated *EPRS* mRNA expression in breast cancers compared to adjacent normal breast, and amplifications of 1q41 involving *EPRS* may drive a subset of breast cancers.

*EPRS* expression has prognostic significance. We found elevated *EPRS* expression to be associated with poorer overall survival in patients with ER+ breast cancers in both TCGA (KM *p* = 5.29e-03, Cox *p* = 1.04e-03; HR = 2.07) and METABRIC datasets (KM *p* = 7.73e-03, Cox *p* = 1.67E-02; HR = 1.23) (Figure 1E). We did not observe a significant association between *EPRS* transcript levels and outcomes for patients with ER^−^ breast cancers (Figure 1F) in either cohort. Thus, *EPRS* may play a role specifically in promoting ER+ tumor growth and treatment sensitivity.

### *EPRS* is associated with tamoxifen resistance

**Figure 2:**
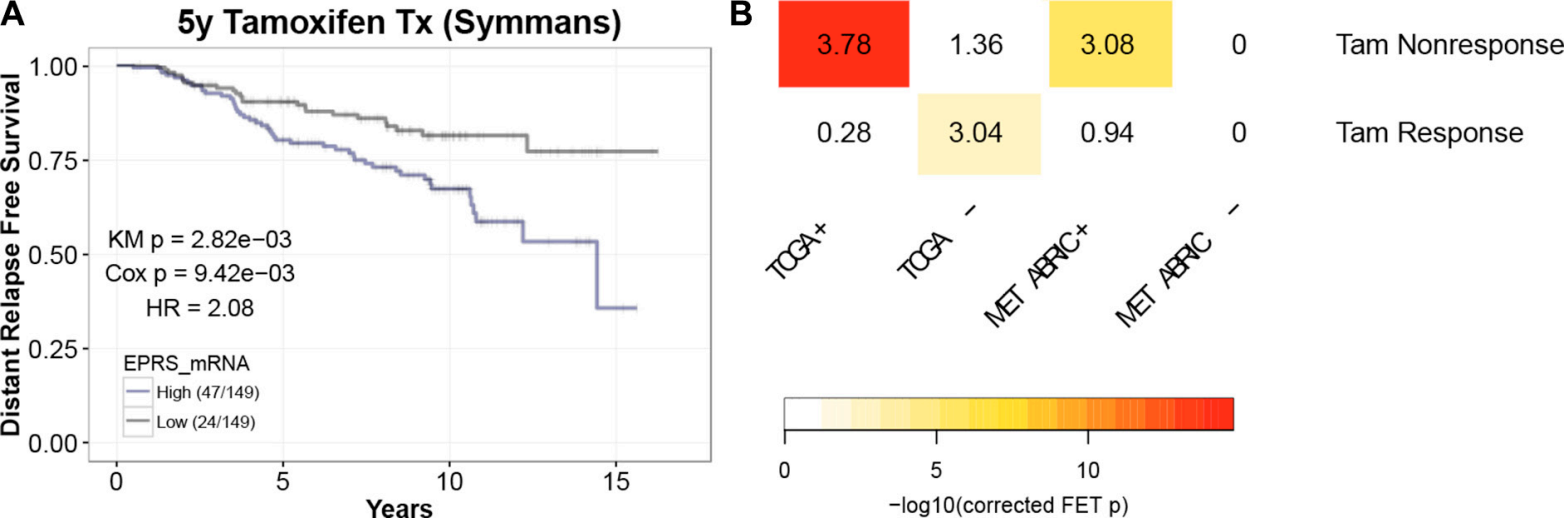
*EPRS* is associated with tamoxifen resistance (**A**) Elevated *EPRS* expression is associated with reduced recurrence-free survival in patients treated with tamoxifen alone for five years (Symmans). (**B**) *EPRS*-correlated genes are enriched for gene signatures of nonresponse to tamoxifen in breast cancer patients. Numbers represent Fold Enrichment.

Endocrine therapies, such as the ER antagonist tamoxifen, are the mainstays of treatment for patients with ER+ breast cancer [12]. Given the prognostic significance of *EPRS* expression for ER^+^ breast tumors and the relatively higher expression of *EPRS* in Luminal B compared to Luminal A tumors, we hypothesized that *EPRS* expression may be associated with reduced sensitivity to endocrine therapy. In a cohort of 298 ER+ breast cancer patients who were treated with tamoxifen alone for 5 years [13], we observed a significant negative association between *EPRS* mRNA expression and recurrence-free survival (KM = 2.82e-03, Cox *p* = 9.42e-03; HR = 2.08) (Figure 2A). The genes whose expression correlates with that of *EPRS* in TCGA and METABRIC ER+ tumor samples are significantly enriched for predictors of nonresponse to tamoxifen therapy [13–15] (TCGA: corrected FET *p* = 3.96-16, FE = 3.78; METABRIC: corrected FET *p* = 2.52-06, FE = 3.08) (Figure 2B). In the TCGA cohort, the genes anticorrelated with *EPRS* are significantly enriched for genes predictive of favorable tumor response to tamoxifen (corrected FET *p* = 1.43e-03, FE = 3.04). These data support a role for *EPRS* in endocrine therapy resistance of ER+ tumors.

### *EPRS* is necessary for proliferation of ER^+^ breast cancer cells

**Figure 3:**
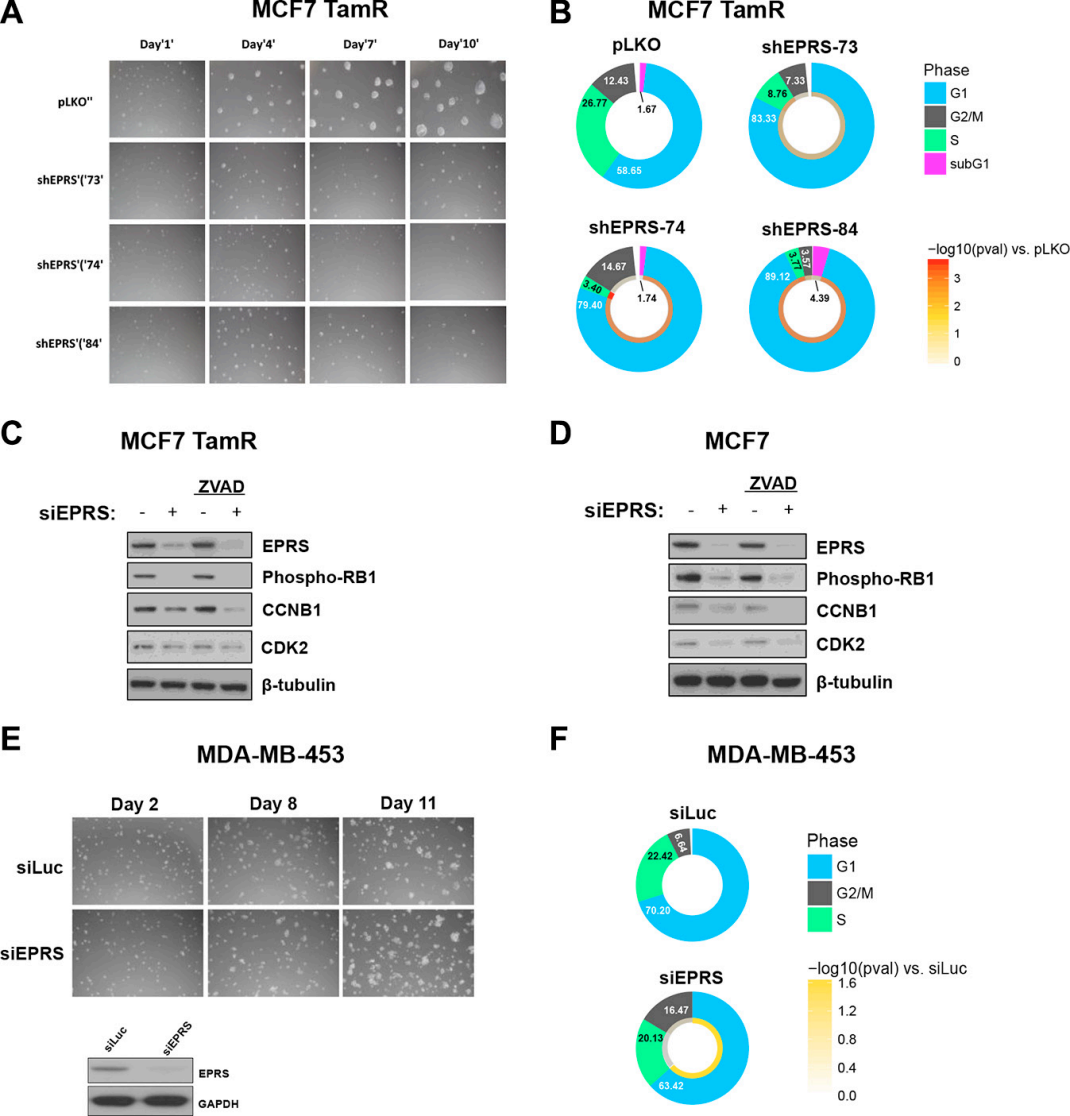
*EPRS* is necessary for proliferation ER+, but not ER− breast cancer cells (**A**) *EPRS* knockdown inhibits growth of MCF7 TamR cells in 3D Matrigel™ culture. (**B**) Flow cytometric analysis of PI-stained DNA. Numbers are mean percentages from three independent experiments. Color ring represents -log10 (student's *t*-test *p* values). C&D) Representative immunoblot of G1/S/G2 proteins in MCF7 TamR (**C**) and parental (**D**) cells treated with *EPRS* or control siRNA in the absence and presence of 20 μM Z-VAD-FMK. (**E**) *EPRS* knockdown does not inhibit growth of ER- MDA-MB-453 cells in 3D Matrigel^™^ culture. Representative immunoblot of *EPRS* knockdown in MDA-MB-453 cells. (**F**) Flow cytometric cell cycle analysis of PI-stained MDA-MB-453 cells. Numbers are mean percentages from three independent experiments. siLuc: luciferase-targeting siRNA (control). siEPRS: pool of four unique siEPRS-targeting siRNAs.

To determine the functional roles of *EPRS* in ER+ breast cancer, we downregulated *EPRS* expression in ER+ breast cancer cell lines using shRNA vectors or siRNA. To determine if *EPRS* is critical for the growth of endocrine therapy-resistant ER+ breast cancer cells, we downregulated *EPRS* in MCF7 TamR cells, an MCF7 variant that was *in vitro* selected for Tamoxifen resistance by continuous culture in the presence of increasing concentrations of Tamoxifen. *EPRS* downregulation inhibited MCF7 TamR growth in 3D Matrigel^™^ cultures (Figure 3A). As the growth inhibition may be due to differences in survival and/or proliferation, we assessed cell cycle progression by flow cytometry. When compared to MCF7 TamR cells expressing vector control shRNA, cells expressing any of three independent *EPRS*-targeting shRNA vectors were found to be arrested in G1 (vector control: 58.65% G1; shEPRS-73: 83.33% G1, *p* = 0.013; shEPRS-74: 79.40% G1, *p* = 2.2E-3; shEPRS-83: 89.12% G1, *p* = 1.9E-3) with a concomitant decrease of cells in S phase (vector control: 26.77%; shEPRS-73: 8.76%, *p* = 5.1E-3; shEPRS-74: 3.40%, *p* = 2.4E-4; shEPRS-84: 3.77%, *p* = 1.2E-3) (Figure 3B). Consistent with these data, we observed downregulation of proteins involved in the G1-to-S and S-to-G2 transition, such as CDK2, CCNB1, and phospho-Rb (Figure 3C, Supplementary Figure S2). We did not observe a consistent increase in the subG1 fraction (Figure 3B) or cleavage of PARP (not shown). Furthermore, *EPRS* depletion induced growth arrest in the presence of the apoptosis inhibitor Z-VAD (Figure 3C). Taken together, these data suggest that apoptosis is not a major mechanism for *EPRS* downregulation-mediated growth inhibition. *EPRS* downregulation similarly affected parental MCF7 cells (Figure 3D). *EPRS* downregulation did not significantly affect the growth of the ER^−^ cell line MDA-MB-453 (Figure 3E and 3F). These data support a critical and specific role for *EPRS* in regulating proliferation of ER+ breast cancer cells.

### *EPRS*-regulated transcriptome

**Figure 4:**
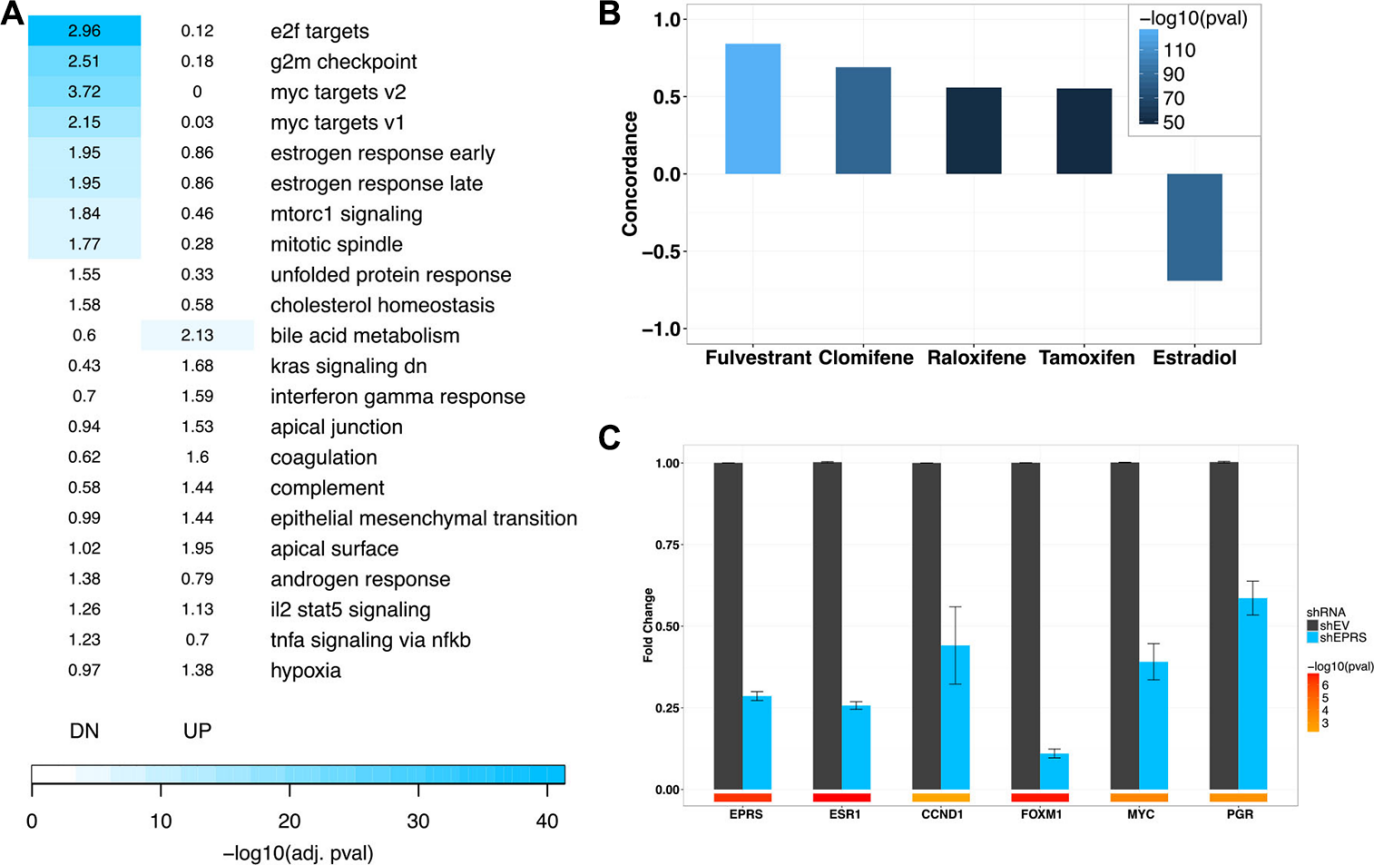
*EPRS*-regulated transcriptome (**A**) Enrichment of shEPRS differentially-expressed genes for MSigDB Hallmark genesets. Numbers represent Fold Enrichment. (**B**) shEPRS differential expression signature overlaps with estrogen receptor modulators. (**C**) QPCR validation of estrogen receptor target downregulation. Error bars represent SEM. Color bars represent -log10 (student's *t*-test *p* values). *EPRS* shRNA-74 shown.

To begin to determine the mechanisms by which *EPRS* regulates G1-S transition and proliferation of ER+ breast cancers, we identified the transcriptional programs regulated by *EPRS* in ER^+^ tumors. We performed paired-end 100-nucleotide Illumina sequencing on MCF7 TamR expressing either vector control or one of three unique *EPRS* shRNA sequences. Using an FDR cutoff of 0.05 and a fold change cutoff of 1.3, we observed 7,129 differentially-expressed genes with shEPRS-73, 10,995 with shEPRS-74, and 5,114 with shEPRS-84 (Supplementary Figure S3). We used genes down- or upregulated by at least two of three shRNA vectors for downstream analysis yielding 3146 downregulated and 3715 upregulated genes (Supplementary Figure S3). We performed functional annotation analysis on this *EPRS* signature using the MSigDB Hallmark gene sets [16]. In agreement with the cell cycle arrest we observed, *EPRS* knockdown downregulated genes involved in cell proliferation: E2F targets (corrected FET *p* = 4.60e-40, FE = 2.96); G2M checkpoint (corrected FET *p* = 2.50e-24, FE = 2.51); Myc targets v1 (corrected FET *p* = 9.80e-21, FE = 3.72); Myc targets v2 (corrected FET *p* = 1.80e-14, FE = 2.15). Consistent with *EPRS*'s known role in inhibiting translation of interferon gamma-induced genes [17] upregulated genes were enriched for interferon gamma response (corrected FET *p* = 0.024, FE = 1.59), as well as bile acid metabolism (corrected FET *p* = 1e-04, FE = 2.13) and genes downregulated by KRAS-signaling (corrected FET *p* = 3e-03, FE = 1.68) (Figure 4A). We did not observe induction of *DDIT3* or downregulation of *COL1A1*, *COL1A2*, or *S100A4*, as has previously been reported in *EPRS*-inhibition-induced stress response [5, 18, 19]. Similarly, enrichment of upregulated genes for “unfolded protein response” was not significant (corrected FET *p* = 0.17, FE = 1.55) (Figure 4A). We did not observe significant upregulation of genes associated with apoptosis (Figure 4A). Interestingly, *EPRS* shRNA expression also strongly downregulated early and late estrogen response genes (corrected FET *p* = 5.90e-10, FE = 1.95) suggestive of a role for *EPRS* in direct regulation of ER signaling (Figure 4A).

### *EPRS* regulates expression of *ESR1* and *ESR1* target transcripts

As *EPRS* knockdown downregulated estrogen response genes, we hypothesized that *EPRS* may regulate cell proliferation through regulation of ER signaling. Using the Broad Institute's Connectivity Map (CMap) [20], we observed striking concordance between gene expression changes induced by *EPRS* shRNA expression and those induced in MCF7 cells by treatment with clinically used anti-endocrine therapies, such as fulvestrant (CMap score = 0.842, *p* = 8.76e-128), clomifene (CMap score = 0.69, *p* = 1.39e-86), raloxifene (CMap score = 0.559, *p* = 6.77e-52), and tamoxifen (CMap score = 0.552, *p* = 3.56e-50) (Figure 4B). We similarly observed a strong inverse association between gene expression changes induced by *EPRS* knockdown and those induced by estradiol treatment of MCF7 cells (CMap score = −0.691, *p* = 6.87e-87) (Figure 4B).

To further validate the link between *EPRS* and ER signaling, we confirmed, by qRT-PCR, that *EPRS* shRNA expression downregulated ERα and the *ESR1* targets *CCND1*, *FOXM1*, *MYC*, and *PGR* (Figure 4C). Furthermore, downregulation of *ESR1* in MCF7 TamR cells, induced growth arrest and prevented phosphorylation of Rb and upregulation of the S/G2 proteins CDK2, and CCNB1 (Supplementary Figure S4A), phenocopying *EPRS* perturbation. This is consistent with persistent dependence of endocrine-resistant breast cancer cells on *ESR1* expression and activity. Based on these results, we hypothesized that reconstitution of *ESR1* expression may rescue the cell cycle arrest we observed with *EPRS* knockdown. Expression of exogenous *ESR1* with *EPRS* siRNA rescued *ESR1* expression and partially rescued siEPRS-induced downregulation of *CCNB1* and phospho-*RB* (Supplementary Figure S4B and S4C), however, it was not able to rescue G1/S arrest by flow cytometry (data not shown).

**Figure 5:**
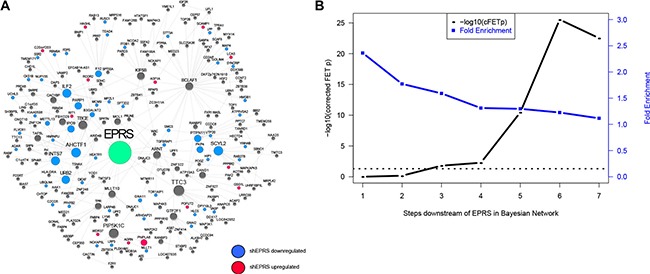
Validation of *EPRS* Bayesian network (**A**) An network neighborhood around *EPRS*. Genes differentially-expressed following *EPRS* knockdown are highlighted in blue (downregulated) or red (upregulated). (**B**) Enrichment of *EPRS* downstream network for the *EPRS*-downregulated RNA-seq signature. Horizontal dashed line represents −log10 (0.05).

Based on these results, we hypothesized that *EPRS* may regulate *ESR1* coactivators and corepressors, as well as other regulators of estrogen signaling. To identify these potential ER regulators in an unbiased manner, we constructed a Bayesian gene regulatory network, as previously described [21], using ER+ breast cancer samples from the TCGA (*n* = 623), METABRIC (*n* = 1505), Miller (*n* = 213) and Wang (*n* = 209) datasets and transcription factor-target interactions from the ENCODE project [22] and MCF7 ChIP-chip data [23]. Bayesian network inference uses directed acyclic graphs (DAGs) to model the joint probability distribution of the states of multiple variables (e.g., genes), characterizing the dependent and independent relationships among genes, which is extremely useful for unraveling gene expression regulatory structures. Bayesian networks constructed from individual datasets were combined by the union of directed edges into a super Bayesian network comprised of 20,810 nodes connected by 73,283 edges. The gold standard for network validation compares a node's network neighborhoods with experimentally-derived perturbation signatures of that node. The *EPRS*-regulated subnetwork is highly enriched for genes downregulated by shEPRS (Figure 5), validating the ability of the Bayesian network to predict gene regulatory relationships. For example, the 5- and 6-layer downstream neighborhoods of *EPRS* are significantly enriched for the *EPRS* knockdown signature with FET *p* values = 3.48e-11 (1.29 fold) and 2.92e-26 (1.23 fold), respectively.

**Figure 6:**
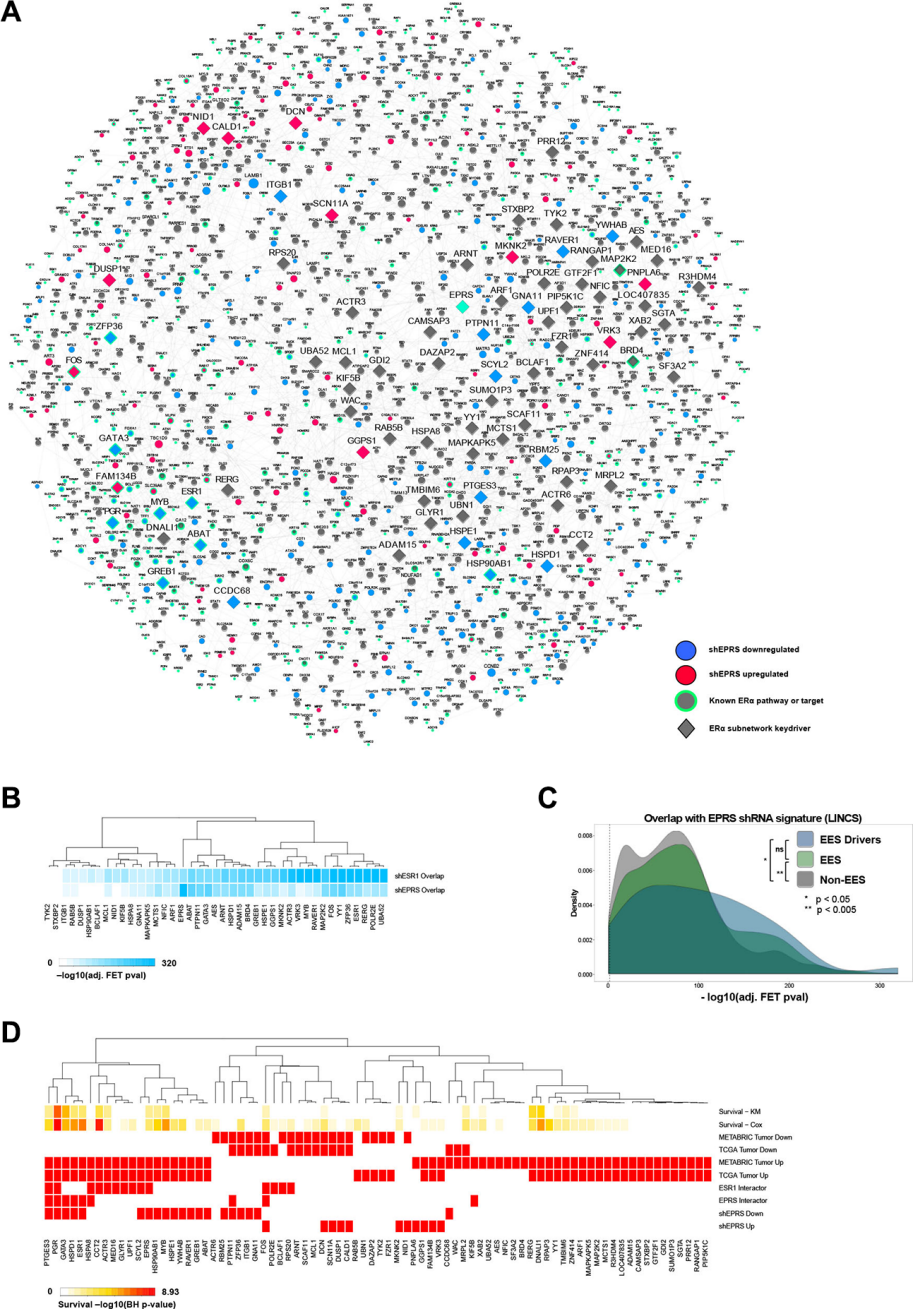
*EPRS*-regulated *ESR1* network (**A**) *EPRS* neighborhood encompassing *ESR1* target and pathway genes. (**B**) Overlap of keydriver shRNA signatures with those of *EPRS* and *ESR1* for genes with perturbations available in the LINCS shRNA dataset. (**C**) As a group, EES driver and EES perturbation signatures show more significant overlaps with the shEPRS signatures than do non-EES genes. (**D**) *EPRS* estrogen network keydriver association with survival using Cox and KM models, differential expression in TCGA and METABRIC ER+ tumors compared to adjacent normal breast, protein interactions with *ESR1* and *EPRS*, and differential expression following *EPRS* knockdown.

To map the direct and indirect regulation of *ESR1* by *EPRS*, we calculated the shortest network paths between *EPRS* and 452 known *ESR1* targets and pathway genes, as described in methods. The genes comprising these paths were then used to derive an *EPRS-ESR1* subnetwork (EES). The EES consists of the 452 known *ESR1* transcriptional target genes and 1079 intermediate nodes connected by 2902 directed edges (Figure 6A).

We performed Key Driver Analysis (KDA) [24, 25] on the EES to identify its master regulators downstream of *EPRS*. This yielded 80 genes (Figure 6) (Supplementary Table S1) predicted to be the drivers of the EES. Many predicted drivers, such as *ESR1* itself, *GATA3, BRD4, PGR*, and *PTGES3* are known regulators of *ESR1* signaling or are *ESR1* effector genes. To confirm that these predicted network drivers are contributing to *EPRS*-mediated regulation of estrogen signaling, we utilized the LINCS dataset to compare the network drivers' perturbation signatures to those of *EPRS* and *ESR1*. For the 41 EES network drivers that had differentially expressed genes in the corresponding perturbation experiments in LINCS their perturbation signatures all significantly overlap with the *EPRS* (corrected FET *p* ≤ 5.04e-04) and *ESR1* (corrected FET *p* ≤ 1.28e-24) signatures (Figure 6B). The overlap between the *EPRS* and *ESR1* LINCS signatures is highly significant (corrected FET *p* = 3.6e-143, OR = 8.86) (Figure 6B). When compared to LINCS shRNA signatures of genes not in the EES, LINCS shRNA signatures of EES keydrivers and of EES genes show significantly greater overlaps with the *EPRS* signature (Kolmogorov-Smirnov *p* = 9.98e-03 and 1.61e-03, respectively) (Figure 6C).

Twenty of the 80 EES drivers are downregulated by shEPRS, and 11 are upregulated (Figure 6D). Nine are previously reported protein-protein interactors of *EPRS* (*HSPA8, ESR1, HSPD1, GATA3, PGR, PTGES3, KIF5B, PTPN11, FOS*) [26], and 14, including *EPRS*, were previously-reported protein-protein interactors of *ESR1* (*EPRS, SCYL2, UPF1, GLYR1, MED16, ACTR3, CCT2, HSPA8, PGR, PTGES3, RP20, BCLAF1, POLR2E, FOS*) [27] (Figure 6D). *HSPA8*, *PGR*, *PTGES3*, and *FOS* are interactors common to both *EPRS* and *ESR1* (Figure 6D). Of the 80 EES drivers, 45 are upregulated in breast tumors compared to adjacent normal breast in both TCGA and METABRIC cohorts, and 61 are upregulated in at least one cohort (Figure 6D). Thirteen are downregulated in both TCGA and METABRIC, and 25 are downregulated in at least one dataset (Figure 6D). Sixteen of the EES drivers are significantly (FDR < 0.10) associated with overall survival in both Cox and KM models, and 43 in at least one model. Furthermore, 34 predicted EES drivers are known ER signaling regulators or downstream targets (Supplementary Table S2), while the remaining 46 have not, to our knowledge, been described in the context of estrogen signaling.

## DISCUSSION

*EPRS* has been previously implicated in breast tumorigenesis, but the underlying mechanisms remained unclear. We have demonstrated that *EPRS* is the most significantly upregulated ARS gene in ER+ breast cancers, partly attributable to *EPRS* copy number gains, and that elevated *EPRS* expression is associated with reduced overall survival in patients with ER+ breast cancers. Higher *EPRS* transcript levels are also associated with reduced distant relapse-free survival in patients treated with adjuvant tamoxifen monotherapy. We further show that *EPRS*-correlated genes are highly enriched for signatures predictive of nonresponse to tamoxifen therapy. Depletion of *EPRS* resulted in mitotic arrest of tamoxifen resistant and parental ER+ breast cancer cells, but not of ER- cells. Transcriptomic profiling confirmed downregulation of cell cycle genes, and showed strong enrichment for downregulation of estrogen response genes. Applying a Bayesian network approach to 2550 ER+ breast cancer samples, we subsequently constructed an *EPRS*-centered directed estrogen signaling network, identified its driver genes, and validated them using publicly-available MCF7 shRNA perturbation signatures. This unbiased approach enabled us to identify 34 known regulators of estrogen signaling, such as *ESR1* itself, *GATA3, BRD4, PGR*, and *PTGES3*, and 46 genes which, to our knowledge, have not been previously associated with endocrine signaling. We have shown these drivers to share strikingly similar perturbation signatures, but further work must be done to assess their functional roles in ER+ breast cancer cells. In addition to gene knockdown and knockout experiments, it will be interesting to see if drugs that reverse the EPRS gene signature are able to inhibit the growth of ER+ breast cancer cells *in vitro* and *in vivo*. We are thus the first, to our knowledge, to identify a regulatory link between *EPRS* and estrogen signaling and the first to provide mechanistic insight into this relationship.

The EPRS depletion phenotype we observed is distinct from that recently reported in the literature. Beltran et al. reported that *EPRS* inhibition by shRNA or the ProRS inhibitor halofuginone (HF) induced a stress response and cell death in basal breast cancer cells [5]. Their work is consistent with another paper in which primary mouse CD4^+^ CD25^−^ cells were treated with HF, resulting in downregulation of *COL1A1*, *COL1A2*, and *S100A4*, and upregulation of *DDIT3* mRNA [18]. Microarray profiling of HF-treated mouse mammary epithelial cells similarly revealed induction of *Ddit3, Trib3, Nrdg1, Gadd45*α*, Slc1a4*, and other genes implicated in cellular response to stress [19]. In contrast, we did not observe differential expression of these genes following *EPRS* knockdown (not shown), suggesting depletion of total *EPRS* protein affects distinct processes compared to specific inhibition of the *EPRS* ProRS domain. An alternate explanation is that ER+ breast cancer cells are more dependent on *EPRS* to maintain ER-mediated growth-promoting signaling. In support of this, *EPRS* depletion in the ER−/HER2+ breast cancer cell line MDA-MB-453 did not inhibit growth. However, we do not discount the possibility of ER signaling-independent mechanisms through which *EPRS* may affect cell growth.

*EPRS* has previously been shown to regulate transcript-specific translation in macrophages and hepatocellular carcinoma cells through the gamma interferon-activated inhibitor of translation (GAIT) system [6, 7, 17, 28–33]. Although GAIT activity has not been demonstrated in other cell types, including breast cancer cells, several genes predicted to be regulated by GAIT, such as *RORA, NRIP1*, and *DZIP3* have been shown to mediate ERα-dependent proliferation of breast cancer cells [34–39].

Further work is required to validate and fully develop the molecular interactome linking *EPRS* to estrogen signaling and cell proliferation in ER+ breast cancer cells. The necessity of *EPRS* in non-malignant cells, as well as the therapeutic window of *EPRS* inhibition must also be determined, although this is beyond the scope of this paper. *EPRS* and the EES it regulates may be a promising target for development of novel therapies, or the repurposing of existing therapies, to treat patients with ER+ breast cancers whose tumors do not respond to currently-used endocrine modulators.

## MATERIALS AND METHODS

### Breast tumor expression data

#### TCGA

The level 3 IlluminaHiSeq-RNASeqV2 (RSEM-normalized) expression data from the TCGA data portal was first log-transformed and quantile-normalized. The quantile-normalized data was then split into the tumor and adjacent normal groups. The tumor and adjacent normal data were then corrected by linear regression for confounding factors including batch, tissue source site, center and plate, race and age. Male samples were excluded.

#### METABRIC

Normalized Illumina HT12v3 mRNA microarray data was downloaded from the European Bioinformatics Institute and corrected for age.

#### Miller and wang

Miller et al. 2005 [40] and Wang et al. 2005 [41] Affymetrix Human Genome U133A array expression datasets were obtained from Bioconductor [42] through the packages “breastCancerUPP” (Miller et al. 2005) and “breastCancerVDX” (Wang et al. 2005) and corrected for covariates as described above.

#### Symmans

Affymetrix Human Genome U133A array expression data was downloaded from the Gene Expression Omnibus (GEO) (GSE17705) and corrected for age and batch.

### CNA analysis

Copy number alterations in the TCGA breast cancer cohort were obtained from the Catalogue of Somatic Mutations in Cancer (COSMIC) [43] and ER+ samples were extracted. METABRIC copy number alterations were called by Curtis et al. 2012 [44]. From the somatic copy number aberration (SCNA) segments called from Curtis *et al*. we first subset segments for ER+ patients (1505 out of 2000 samples combining discovery and validation data sets), and counted the number of segments called with copy number gain (denoted as GAIN) or amplification (denoted as AMP) within 2Mbps of transcription starting site of *EPRS* from hg18 (NCBI build 36).

### Survival analysis

*EPRS* expression from the previously described TCGA, METABRIC, and Symmans breast cancer cohorts, was split into ER+ and ER- groups. Within each ER status-specific *EPRS* profile, we defined “*EPRS*-high” and “*EPRS*-low” subgroups of patients by median *EPRS* expression value. We performed the Kaplan-Meier analysis for *EPRS*-high and *EPRS*-low in both ER+ and ER- cohorts as well as Cox regression analysis to evaluate the prognostic significance of the subgroups. Correction for multiple hypothesis testing was done using the Benjamini-Hochberg (BH) method [45].

### Tamoxifen response signature

A tamoxifen response/nonresponse signature was identified by combining published gene signatures predictive of response [13–15]. Our final signature consisted of 364 genes comprised of 124 predictive of tamoxifen response and 240 predictive of tamoxifen nonresponse (Supplementary Table S1).

### *EPRS*-correlated genes

Spearman correlations between *EPRS* and all other genes in TCGA and METABRIC datasets were computed and *p*-values were corrected using the BH method [45]. Significant correlations were defined by BH corrected *p*-value < 0.05 and absolute value of Spearman's rho > 0.2.

### Quantitative real-time PCR

Total RNA was isolated from cells using the Qiagen RNeasy kit. RNA concentration was determined using a NanoDrop 8000 spectrophotometer. Fifty nanograms of total RNA were used per triplicate qRT-PCR reaction and detected using SYBR Green fluorescence. *GAPDH* was used as an internal control. QRT-PCR data was analyzed using the delta-Ct method.

### Cell culture

MCF7 parental cells were cultured in RPMI media with 10% FBS (Life Technologies). MCF7 TamR cells, obtained from the Rachel Schiff Lab (Houston, TX) [46, 47], were cultured in phenol red-free RPMI media (Life Technologies) with 10% charcoal-stripped fetal bovine serum (FBS) (Sigma), one percent penicillin/streptomycin (Life Technologies), and 100 nM 4-hydroxytamoxifen (4-OHT). 4-OHT was withdrawn for functional assays. MDA-MB-453 cells were cultured in DMEM media (Life Technologies) with 10% FBS (Life Technologies) and one percent penicillin/streptomycin (Life Technologies).

### 3D Matrigel^™^ cultures

Eight-well chamber slides (BD Biosciences) were coated with Matrigel^TM^. Cells were plated at a density of 3–5 × 10^3^ cells per well in the middle four wells, and cells were allowed to grow for two to three weeks.

### RNAi

For siRNA, cells were reverse-transfected with 20 nM siRNA using Lipofectamine RNAiMAX (Life Technologies) according to the manufacturer's protocol. Media was changed after overnight incubation. *EPRS* shRNAs in the pLKO.1 vector were obtained from Sigma. For shRNA lentivirus production, 293T cells were transfected with viral plasmids using Lipofectamine 2000 according to standard protocols. Virus-containing supernatant was collected at 48, 72, 96, and 120 hours after transfection, pooled, and frozen at −80°C to eliminate carry-over 293T cells. For shRNA lentivirus infection of target cells, lentivirus was thawed on ice and concentrated using centrifugal filters (Amicon). ShRNA lentivirus and target cells were simultaneously added to six-well plates, centrifuged at 2250 rpm for 30 minutes at room temperature, then incubated at 37°C overnight. Media was changed the next morning and cells were allowed to recover for six to eight hours, after which 2 μg/mL puromycin was added to select shRNA-expressing cells. Puromycin was reduced to a maintenance dose of 1 μg/mL after 24–48 h. For Z-VAD-FMK (BD Biosciences; San Jose, CA) treatment, cells were treated with 20 μM Z-VAD-FMK or DMSO.

### Cell cycle analysis

Cells were infected with shRNA or transfected with siRNA as described above. Twenty-four hours after siRNA transfection or 48 hours after shRNA transfection and puromycin selection, cells were serum-starved in serum-free growth media for 24 hours, after which serum was reintroduced and cells are incubated for an additional 48 hours. Five days after RNAi introduction, cells were trypsinized, split, and lysed for total protein for Western blot analysis or fixed in 80% ethanol and stored at 4°C for flow cytometric analysis. Cells were then stained with propidium iodide/RNAase (BD Pharmigen) for 30 minutes at 37°C and analyzed on a BD FACSCanto flow cytometer. Cell cycle analysis was performed with FlowJo (TreeStar Inc) using both Watson-Pragmatic [48] and Dean/Jett/Fox [49] algorithms.

### RNA-seq analysis

Cells expressing *EPRS* shRNA vectors were generated. Seventy-two hours after infection, cells were lysed and total RNA was purified using a Qiagen RNeasy kit according to the manufacturer's protocol. RNA was stored at −80°C until submission to the Mount Sinai Genomics Core Facility for ribosomal RNA depletion, cDNA library preparation, and sequencing using paired-end, 100 nt reads on an Illumina HiSeq 2000. The pair-ended sequencing reads were aligned to human genome hg38 using star aligner (version 2.5.0 b) [50]. Following read alignment, featureCounts [51] was used to quantify gene expression at the gene level based on GENCODE gene model release 22. Gene expression was normalized as counts per million (TPM) using trimmed mean of M-values normalization (TMM) method [52] to adjust for sequencing library size difference. Differential gene expression was predicted using the Bioconductor package limma [53]. The false discovery rate (FDR) of the differential expression test was estimated using the Benjamini–Hochberg method. We used a cutoff of corrected *p* < 0.05 and fold change > 1.3 in at least two of three *EPRS*-targeting shRNAs. Overlap between independent *EPRS*-targeted shRNAs was tested for statistical significance and visualized using the SuperExactTest [54]. Total RNA used for RNA-seq was subsequently used for validation by qRT-PCR.

### Connectivity map query

The differentially expressed genes (DEGs) between the shEPRS and control samples were compared with the drug treatment gene expression data from the Connectivity MAP (CMap) database, a reference collection of gene expression data from 5 cultured human cell lines treated with 1309 drug compounds [55]. In the original CMap study, the Kolmogorov-Smirnov (KS) statistic was employed to compute the similarity between an input DEG signature and a drug's gene signature. A significant KS statistic suggests some functional connection between drug treatment and the biology represented by a given input DEGs [55]. In this study, we utilized a weighted KS test which was previously proposed for gene set enrichment analysis [56] to calculate the drug connectivity score. We described the analytic approach below.

Let *R* be the *N* genes which are rank sorted in descending order of the expression fold change in a CMap drug instance. Next the ranks are converted to weighted gene-drug correlations using a formula $r=\frac{\text{Rank}-\text{mean}(\text{Rank})}{\text{mean}(\text{Rank})}$, through which the genes ranked in the top or bottom (i.e. the most induced or suppressed) had absolute drug correlation scores close to 1, while genes ranked in the middle (possibly the least targeted by the drug) had gene-drug correlation measures close to 0. Let *G* be a set of *n* input DEGs, which is comprised of two subsets, *G*_1_ and *G*_2,_ representing the significantly up- and down-regulated genes, respectively. For each subset *G*_x_ (*x* = 1 or 2), we then compute a drug connectivity scores *S*_x_ as the maximum derivation from zero, i.e., *f*_1_(*i*) − *f*_2_(*i*), where *i* (= 1, …, *N*) indices a position in the rank of *R*, and

$$\begin{array}{l} f_{1}(i)=\sum_{\begin{array}{l} g_{j}\in G_{x}\\ j\leq i \end{array}}\frac{|r_{j}{|}^{p}}{M},\text{where }M=\sum_{g_{j}\in G_{x}}|r_{j}{|}^{p},\\ \;f_{2}(i)=\sum_{\begin{array}{l} g_{j}\notin G_{x}\\ j\leq i \end{array}}\frac{1}{N-n_{x}} \end{array}$$

Here, j is an index with a value between 1 and i, and *g_j_* denotes the *j*th gene in the rank sorted list *R*. Finally, the drug connectivity score *S* = 0 if *S*_1_ × *S*_2_ ≥ 0, and *S* = *S*_1_ − *S*_2_ otherwise. When *p* = 0, this method reduces to standard KS test. We set *p* = 1 for the analyses in this paper.

The statistical significance of *S* can be estimated from permutation. The sign of *S* reflects the direction of the drug treatment with respect to the input gene signatures. A positive *S* implies that the drug has a gene signature that is concordant to the input signature and that drug could induce the same transcriptional regulation pattern of the input genes, while a negative *S* implies that the drug has a gene expression signature that is opposite to the input signature and the drug could potentially reverse the disease trait.

### Bayesian network construction

We employed a Monte Carlo Markov Chain (MCMC) simulation process based approach [57] to infer probabilistic regulatory relationship between genes. A uniform prior was used for the regulatory relation between pair of genes. As a uniform prior is unable to break Markov equivalence, we need additional data to assist with the identification of directionality in the Bayesian network construction. For this purpose, known transcription factor (TF)-target pairs were downloaded from the ENCODE project [22] and a dataset of nuclear receptor binding sites in breast cancer cells [58]. We allowed TF nodes to be parent nodes of their targets, but targets were not allowed to be the parent nodes of their TF in the network. As in [57], we followed a network averaging strategy in which 1,000 networks were generated by this MCMC process starting with different random structure, and links that appeared in more than 30% of the networks were used to define a final consensus network. If loops were present in the consensus network, the weakly supported link involved in a loop was removed to ensure the final network structure was a directed acyclic graph. Bayesian networks constructed from independent datasets were then combined by the union of directed edges, and loops were removed.

### *EPRS* subnetwork

Estrogen signaling pathway gene sets were obtained from the Pathway Interaction Database (ERalpha pathway) [59], MSigDB Hallmark gene sets (Estrogen response early; Estrogen response late) [16], KEGG (Estrogen Signaling) [60], Gene Ontology (Intracellular estrogen receptor signaling pathway) [60], and recent literature findings [61–65] for a total of 452 unique estrogen signaling genes (ESG). The shortest paths between *EPRS* and all genes in ESG in the super Bayesian network were computed. *EPRS*, ESGs, and all intermediate connecting nodes were called the *EPRS-ESR1* subnetwork (EES). Keydriver Analysis (KDA) was performed on the EES as previously described [24].

### LINCS differential expression signature calling

Normalized level 3 expression data was downloaded from the Library of Integrated Network-based Cellular Signatures (LINCS) cloud website (http://www.lincscloud.org/l1000/). LINCS data includes the directly measured expression levels of 978 landmark genes and the inferred expression levels of more than 21 K other genes. Gene expression profiles of both directly measured landmark transcripts and inferred genes were normalized using an 80-gene invariant set scaling followed by the quantile-normalization. Differentially-expressed gene signatures for each shRNA were called using the *limma* R package based on the criteria of significance *p* < 0.05 and fold-change > 1.2.

### Reagents

#### Antibodies

*EPRS* (Abcam ab31531); *ESR1* (Cell Signaling 8644); phospho-*RB* (Cell Signaling 9301); total *RB* (Cell Signaling 9309); *CDK2* (Cell Signaling 2546); *CCNB1* (Cell Signaling 12231); *GAPDH* (Cell Signaling 2118), *β-tubulin* (Cell Signaling 2128), *HSP70* (Cell Signaling 4872).

#### QPCR primers

*EPRS* (sense: 5′ –GCCTTCAGGGACAGTAAGCA – 3′, antisense: 5′ – ATGAAGTTGCTGCACAAGGG – 3′); *ESR1* (sense: 5′ – AGAGGGTGCCAGGCTTTGT – 3′, antisense: 5′ – CAGACGAGACCAATCATCAGG – 3′); *CCND1* (QuantiTect Primer Assay # QT00495285, Qiagen); *MYC* (sense: 5′ – CACCGAGTCGTAGTCG AGGT – 3′, antisense: 5′ – TTTCGGGTAGTGGAAAA CCA – 3′); *PGR* (sense: 5′ – AGCCAGAGCCCACAAT ACAG – 3′, antisense: 5′ – GACCTTACAGCTCCC ACAGG – 3′); *FOXM1* (sense: 5′ –TGCAGCTAGGGATGT GAATCTTC – 3′, antisense: 5′ - GGAGCCCAGTCCAT CAGAACT – 3′).

#### RNAi

*EPRS* and *ESR1* siGENOME SMARTpool^™^ pools of four siRNA sequences (Dharmacon GE Healthcare). shRNA: pLKO-empty vector, shEPRS-73 (TRCN0000293873, 5′ – CCGGGCCAAGTACTACACCT TATTTCTCGAGAAATAAGGTGTAGTACTTGGCTT TTTG – 3′), shEPRS-74 (TRCN0000293874, 5′ – CCG GATGAACCTGTTAGCCCATATACTCGAGTATATGG GCTAACAGGTTCATTTTTTG – 3′), and shEPRS-84 (TRCN0000286384, 5′ - CCGGGCCTGGCAAGAACAG TTGAAACTCGAGTTTCAACTGTTCTTGCCAGGCTT TTG - 3′).

#### Cell lines

Parental and TamR MCF7 cells were obtained from the Rachel Schiff Lab (Baylor College of Medicine, Houston, TX) [46, 47]; MDA-MB-453 cells were purchased from ATCC (Manassas, VA).

#### Exogenous ESR1-expressing MCF7 TamR cell line

*ESR1* cDNA was obtained from Addgene (#11351) and was cloned into pBABE-puro. MCF7 TamR cells were stably infected with empty vector (EV)- or *ESR1*-pBABE-puro retrovirus and selected as described above.

## SUPPLEMENTARY MATERIALS






