# Supplementary material for: EPRS is a critical regulator of cell proliferation and estrogen signaling in ER+ breast cancer
Source: Oncotarget. 2016 Sep 6;7(43):69592–605. doi: 10.18632/oncotarget.11870 (PMC5342500; doi:10.18632/oncotarget.11870)
Supplement: Supplementary file 1 [file oncotarget-07-69592-s001.pdf]

EPRS is a critical regulator of cell proliferation and estrogen signaling in ER+ breast cancer

Supplementary Materials

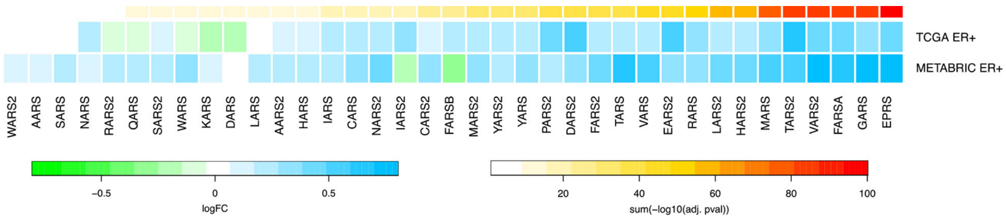

Supplementary Figure S1: Aminoacyl tRNA synthetase expression in ER+ breast cancer. Differential expression of ARS genes in ER+ breast tumors compared to adjacent normal samples in TCGA and METABRIC cohorts. Genes are ordered by the sum of their –log10(adjusted *p* values) from each individual dataset.

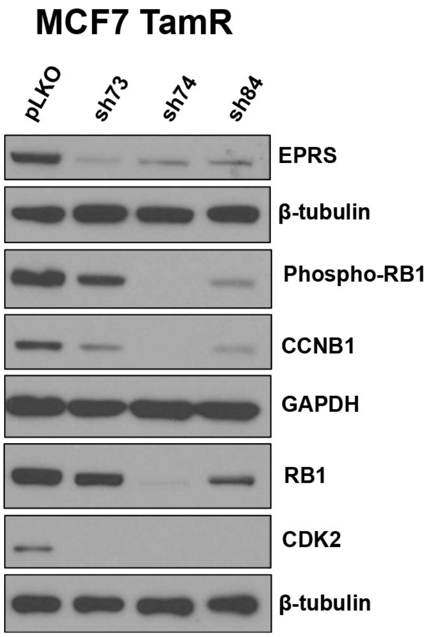

Supplementary Figure S2: *EPRS* shRNA downregulates cell cycle proteins in MCF7 TamR cells. Representative western blot of shEPRS-induced downregulation of cell cycle proteins in MCF7 TamR cells.

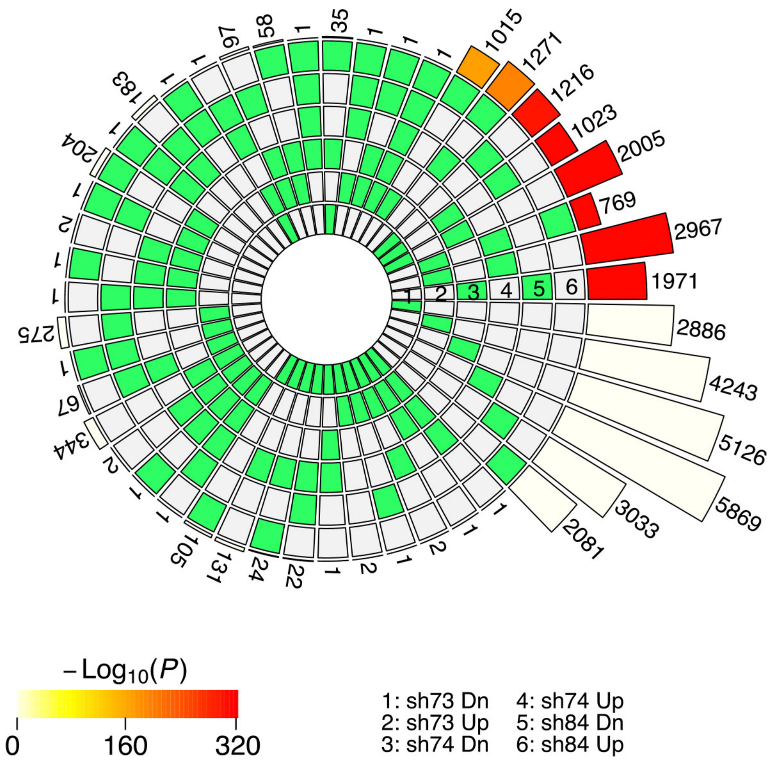

Supplementary Figure S3: shEPRS RNA-seq. *EPRS* shRNA RNA-sequencing differentially-expressed genes (FDR < 0.05, FC > 1.3). Overlaps between shRNAs are tested and visualized using the SuperExactTest.

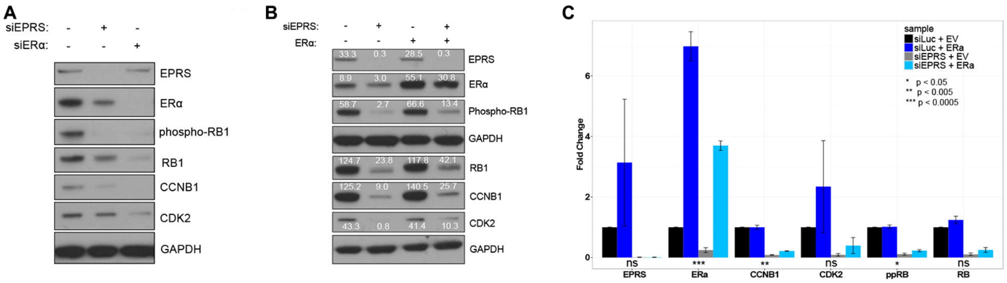

Supplementary Figure S4: *EPRS* affects proliferation partially through *ESR1*. (**A**) *ESR1* knockdown phenocopies *EPRS* knockdown. Representative immunoblot shown. (**B** and **C**) Rescue of *ESR1* expression partially rescues siEPRS-induced downregulation of cell cycle proteins. Error bars represent SEM. siLuc: luciferase-targeting siRNA (control). siEPRS: pool of four unique siEPRS-targeting siRNAs.

Supplementary Table S1: Tamoxifen response and nonresponse genes.
See Supplementary_Table_S1

Supplementary Table S2: EES keydrivers. “1” indicates affirmative, “0” indicates negative. “Survival.cox” and “survival.km” indicate Cox regression and Kaplan-Meier, respectively, *p*-values combined from TCGA, METABRIC, Miller and Wang datasets using Fisher’s method.
See Supplementary_Table_S2
